# Reliability of Measured Data for pH Sensor Arrays with Fault Diagnosis and Data Fusion Based on LabVIEW

**DOI:** 10.3390/s131217281

**Published:** 2013-12-13

**Authors:** Yi-Hung Liao, Jung-Chuan Chou, Chin-Yi Lin

**Affiliations:** 1 Department of Information Management, Transworld University, 1221 Zhennan Rd., Yunlin 64063, Taiwan; 2 Graduate School of Electronic and Optoelectronic Engineering, National Yunlin University of Science and Technology, 123 University Rd., Yunlin 64002, Taiwan; E-Mails: choujc@yuntech.edu.tw (J.-C.C.); m10113316@yuntech.edu.tw (C.-Y.L.)

**Keywords:** fault diagnosis, data fusion, ruthenium dioxide, sensor array, LabVIEW

## Abstract

Fault diagnosis (FD) and data fusion (DF) technologies implemented in the LabVIEW program were used for a ruthenium dioxide pH sensor array. The purpose of the fault diagnosis and data fusion technologies is to increase the reliability of measured data. Data fusion is a very useful statistical method used for sensor arrays in many fields. Fault diagnosis is used to avoid sensor faults and to measure errors in the electrochemical measurement system, therefore, in this study, we use fault diagnosis to remove any faulty sensors in advance, and then proceed with data fusion in the sensor array. The average, self-adaptive and coefficient of variance data fusion methods are used in this study. The pH electrode is fabricated with ruthenium dioxide (RuO_2_) sensing membrane using a sputtering system to deposit it onto a silicon substrate, and eight RuO_2_ pH electrodes are fabricated to form a sensor array for this study.

## 1. Introduction

In the electrochemical field, sensors are the primary devices used for data acquisition. If the sensor shows performance degradation or fails, it will have a serious effect on the measurement or monitoring process. Tomchenko *et al.* [1] reported a sensor array consisting of discrete thick-film sensors based on various semiconductor metal oxides and the sensor array was used for the selective detection of combustion gases. Zhu *et al.* [2] proposed a model based on principal component analysis and a neural network for the multi-fault diagnosis of sensor systems. By means of data fusion [3–5], different sources of information are combined to improve the performances of the system. Fusion may be useful for several objectives such as detection, recognition, identification, tracking, change detection, decision making, *etc*. These objectives may be encountered in many application domains such as defense, robotics, medicine, electrochemistry, *etc*. Kewley [6] introduced the notion that in data fusion the simple form is data + algorithms + knowledge equal to data fusion. Xie and Quan [7] reported that the data fusion method could be applied in the fault diagnosis field. The faults are diagnosed through three levels which are data fusion level, feature level and decision level respectively. Nassar and Kanaan [8] surveyed and discussed the state-of-the-art studies related to the factors affecting the performance of data fusion algorithms, and have integrated data fusion performance research findings. Xue [9] presented a fault diagnosis system based on multi-sensor data fusion algorithm, which is composed of a local data fusion level and whole data fusion level. In order to measure a physical quantity, a sensor is defined as a measuring device that exhibits a characteristic of an electrical nature (such as charge, voltage and current). In electrochemical measurements this consists of a prepared sensing device as a working electrode, and a reference electrode. These electrodes are enclosed in the sensor housing in contact with a liquid electrolyte. The measured pH value is a very important parameter in many fields, such as wastewater monitoring, clinical diagnosis and culture. The pH sensor array fabricated by using ruthenium dioxide thin film with sputtering has been investigated [10]. Zhang *et al.* [11] introduced several kinds of methods for sensor fault diagnosis technology. Xu *et al.* [12] proposed a method of sensor fault diagnosis based on the least squares support vector machine online prediction.

This paper utilizes a fault diagnosis method and integrates some data fusion algorithms to apply them to a pH sensor array. We used the fault diagnosis to obtain the coefficients of the confidence matrix and to judge faulty sensors, and then the measured pH data of the faulty sensor are eliminated. The measured pH data of the other sensors are then used in the average, self-adaptive and coefficient of variance data fusion methods to perform data fusion. The pre-processing of the measured data before data fusion can increase the reliability of pH measurement results. Therefore, the primary objective of this paper was to investigate and compare the reliability of measured pH data after applying fault diagnosis technology with data fusion algorithms implemented in LabVIEW.

## 2. Experimental Section

### 2.1. Material and Reagents

Silicon wafer was used as the substrate of the ruthenium dioxide (RuO_2_) sensing membrane of the sensor device. The silicon substrate was (100)-oriented, p-type, resistivity 15∼25 Ω-cm, and supported by the National Nano Device Laboratories (NDL). The RuO_2_ sensing membranes were prepared using a sputtering process. The RuO_2_ thin films were_deposited on the silicon substrate maintained at 25 °C by radio frequency sputtering with 2-inch-diameter, ¼ in.-thickness, and 99.99% purity ruthenium target. Ethanol and D.I. water were used for cleaning the silicon substrate and were obtained from Katayama (Osaka, Japan) and our laboratory, respectively.

### 2.2. Preparation of Ruthenium Dioxide pH Electrode Array

**Figure 1.**
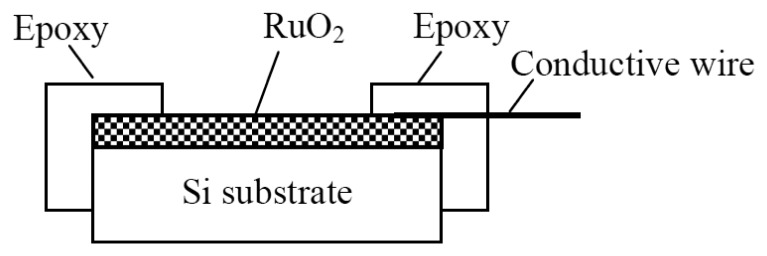
Cross-section of the ruthenium dioxide pH electrode.

The silicon substrates were alternately cleaned ultrasonically in ethanol and D.I. water for 15 min, leached in distilled water, and then dried. In this experiment, the sputtering total operating pressure of 10 mTorr in Ar-gas-mixed O_2_ for 1 h was achieved, the gas flow ratio of the Ar:O_2_ was 4:1 (in sccm), and the radio frequency power was 100 W, at 13.56 MHz. The ruthenium dioxide thin films were obtained from the sputtering system. In the sensing structure, we used the RuO_2_ membrane as sensor head and encased it in epoxy, leaving a 2 mm × 2 mm sensing window as sensing region. The cross-section of the resulting ruthenium dioxide sensing membrane is shown in Figure 1. The eight RuO_2_ pH electrodes form a pH sensor array and were applied to measure the pH values of commercial drinks such as grape wine, coca cola and water, *etc*.

### 2.3. Measurement Set-up

**Figure 2.**
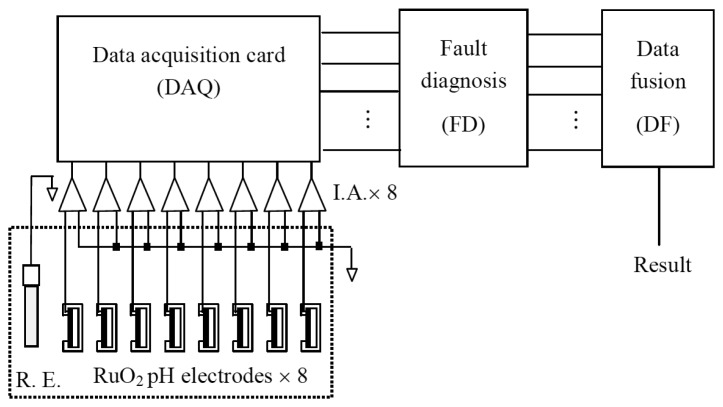
Measurement system used for the ruthenium dioxide pH sensor array with a data acquisition card to acquire data. The data were used to perform fault diagnosis and data fusion.

In this work, we employed eight sensors with ruthenium dioxide sensing membranes as a pH sensor array. In the experimental process the pH sensor array and a Ag/AgCl reference electrode were immersed in grape wine, coca cola and water, respectively. The Ag/AgCl reference electrode provided a stable potential in the measurement process. The schematic diagram of the measurement system is shown in Figure 2.

This measurement system is composed of eight instrument amplifiers (IAs), a data acquisition card, a potentiometric sensor (as working electrode, WE) and a reference electrode (RE) to be immersed in the solutions and can obtain the difference of voltage between working electrode and reference electrode by means of the digital mulii-function meter (HP34401A).

### 2.4. Fault Diagnosis and Data Fusions

In electrochemistry the measurement results are obtained from sensors. If the sensors are degraded, faulty or fail in the measurement and monitoring process this will have serious effects. Therefore, the sensor fault diagnosis is very important in any measurement system. We assume that the output of sensors are u_1_, u_2_,…, u_n_ and the variance of output values are $\sigma_{1}^{2}$, $\sigma_{2}^{2}$,…, $\sigma_{n}^{2}$, then we can obtain the consistent (*d_ij_*) of *i*^th^ sensor and *j*^th^ sensor as follows [13]:

(1) $$d_{ij}=\exp\left[-\frac{1}{2}\cdot \frac{{\left(u_{j}-u_{i}\right)}^{2}}{{\left(2\sigma_{i}\right)}^{2}}\right]$$

The confidence matrix is obtained from Equation (1) from the confidence of each sensor and the confidence matrix was as described by Equation (2). From the coefficients of confidence matrix, we can detect any fault sensors among the pH sensor array:

(2) $$D=\left(\begin{array}{cccc} d_{11} & d_{12} & \cdots & d_{1n}\\ d_{21} & d_{22} & \cdots & d_{2n}\\ \vdots & \vdots & \ddots & \vdots \\ d_{n1} & d_{n2} & \cdots & d_{nn} \end{array}\right),$$

where n is the sensor number.

**Figure 3.**
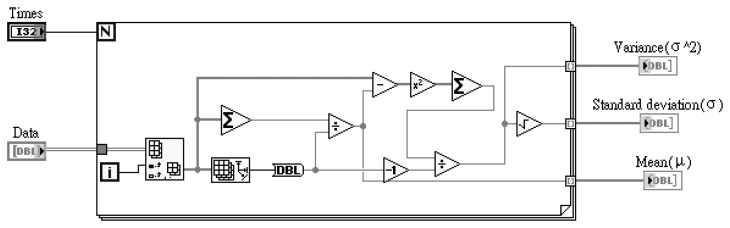
Block diagram of LabVIW for pre-calculation with measured data of the sensor array.

**Figure 4.**
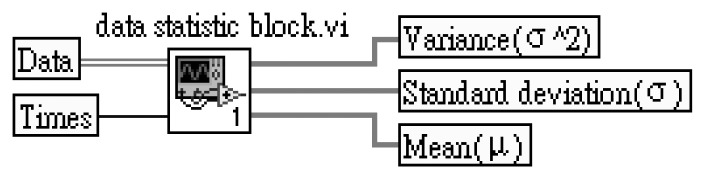
Data statistic block integrated block diagram of Figure 3 with variance, standard deviation and mean.

In this study, the average data fusion (ADF), self-adaptive data fusion (SADF) and coefficient of variance data fusion (CVDF) are used for the ruthenium dioxide-based electrochemical sensor array. These data fusion technologies are designed using LabVIEW software, purchased from National Instrument (NI) Co. Ltd. The pre-calculation of mean, standard deviation and variance are from measured data before data fusion and the designed block diagram is as shown in Figure 3:

The mean (μ), standard deviation (σ) and variance (σ^2^) parameters are obtained from the LabVIEW block diagram. The LabVIEW program of Figure 3 is integrated and named “data statistic block.vi”. The data statistic block.vi program diagram is shown in Figure 4.

In this study, we applied three data fusion methods to the measured data from the pH sensor array. The average data fusion (ADF) is the easiest data fusion method; it has the same weighted coefficients for the pH electrode array. We denote that a set of pH data from the *i*^th^ pH sensor is x = x_1_, x_2_, …,x_n_. The average of the measured data is typically defined as *x̄* The average of the pH data of the *i*^th^ pH sensor is used to calculate it using the following equation [10]:

(3) $${\bar{x}}_{i}=\frac{1}{n}\sum_{i=1}^{n}x_{i}$$

The weighted coefficients *(w_ADF,i_)* of the average data fusion are obtained from Equation (4) and are as follows:

(4) $$w_{\text{ADF},i}=\frac{1}{n},\text{n is number of sensor}$$

We assumed that the pH sensor array has eight pH sensors. We evaluated the weighted coefficients *w_ADF,i_* (*w_ADF,1_, w_ADF,2_*, … *w_ADF,8_*) for each pH sensor and the sum of weighted factors for each pH sensor is equal to unity. The final fusion result with pH sensor array is obtained from the following equation [10]:

(5) $$X_{\text{ADF}}=\sum_{i=1}^{8}w_{\text{ADF},i}{\bar{x}}_{i}$$

**Figure 5.**
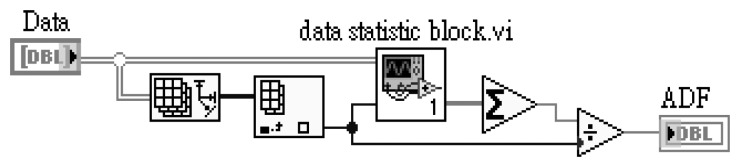
LabVIEW block diagram of average data fusion used for RuO_2_ based pH sensor array.

The fusion function of average data fusion is designed in LabVIEW program for pH sensor array and the block diagram is shown in Figure 5.

We utilize the sensor array based on the minimum mean variance to perform data fusion. First, we assume that all data of each sensor have the same mean and exclusion independent each other. We evaluated the weighted coefficients w_i_ (w_1_, w_2_, … w_n_) for each sensor and the sum of weighted factors for each sensor is equal to unity. The estimated data fusion value μ_y_ can be described as follows [10]:

(6) $$\mu_{y}=\frac{1}{n}\sum_{i=1}^{n}y_{ij},(j=1,2,\ldots ,n)$$

The variances of measured data are obtained from the pre-calculation block diagram of LabVIEW. The expressed equation for *w_SADF,i_* is obtained as follows [10]:

(7) $$w_{S\text{ADF},i}=\frac{1}{\sigma_{i}^{2}\left(\overset{n}{\underset{k=1}{\text{∑}}}\sigma_{k}^{-2}\right)},(i=1,2,\ldots ,n)$$

We evaluated the weighted coefficients *w_SADF,i_ (w_SADF,1_, w_SADF,2_, … w_SADF,n_)* for each sensor and the sum of weighted factors for each sensor is equal to unity. The estimated data fusion value y can then be described as follows [10]:

(8) $$X_{\text{SADF}}=\sum w_{\text{SADF},i}\mu_{i}$$

**Figure 6.**
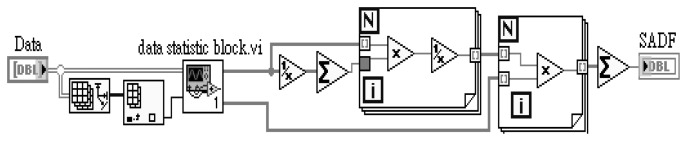
LabVIEW block diagram of self-adaptive data fusion used for RuO_2_ based pH sensor array.

The fusion function of self-adaptive data fusion is completed in LabVIEW program for sensor array and the block diagram is shown in Figure 6.

The coefficient of variance (CV), also named discrete coefficient, is used for different measurement data. The CV is the ratio of the standard deviation and mean value. The CV_i_ is presented as the coefficient of variance of measured data X_i_, and the calculation of the CV_i_ is described as follows [13]:

(9) $$CV_{i}=\frac{\sigma_{i}}{\mu_{i}}$$

The coefficient of variance is used to obtain the weighting coefficients for pH sensor array and is determined as follows [13]:

(10) $$w_{\text{CVDF},i}=CV_{i}^{-1}/\sum_{j=1}^{n}CV_{j}^{-1},i=1,\ldots ,n$$

We evaluated the weighted coefficients *w_CVDF,i_ (w_CVDF,1_, w_CVDF,2_, … w_CVDF,n_)* for each sensor and the sum of weighted factors for each sensor is equal to unity. The sensor array utilizes the above weighted coefficients to derive the fusion result, which is described as follows [13]:

(11) $$X_{\text{CVDF}}=\sum w_{\text{CVDF},i}\mu_{i}$$

**Figure 7.**
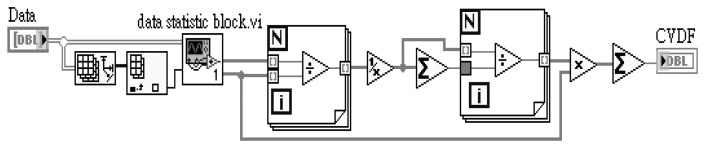
LabVIEW block diagram of coefficient of variance data fusion used for RuO_2_ based pH sensor array.

The fusion function of coefficient of variance data fusion is implemented in LabVIEW program for pH sensor array and the block diagram is shown in Figure 7.

## 3. Results and Discussion

### 3.1. Sensing Characteristics of RuO_2_ Sensor Array

**Figure 8.**
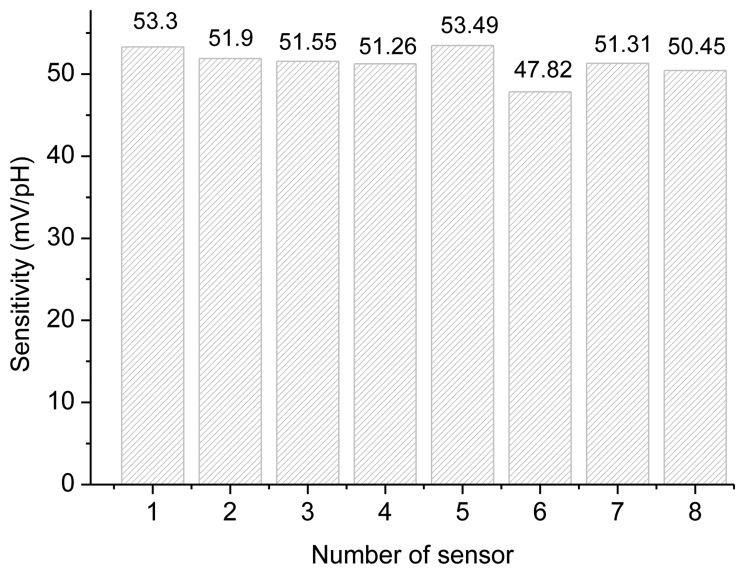
Sensitivity characteristics of ruthenium dioxide pH sensor array from pH 1 to pH 13 buffer solutions.

The sensing characteristics of the ruthenium dioxide pH sensor array in standard buffer solutions were investigated. We use current-voltage measurements to extract the sensitivity of the RuO_2_ sensor array. We used the RuO_2_ sensor array as working electrodes, the Ag/AgCl as reference electrode. Both the working electrode and Ag/AgCl reference electrode were immersed in standard buffer solutions from pH 1 to pH 13. According to experimental results, the average sensitivity is 51.39 mV/pH, and the sensitivities of each sensor are between 47.82 mV/pH and 53.49 mV/pH, which are shown in Figure 8.

**Figure 9.**
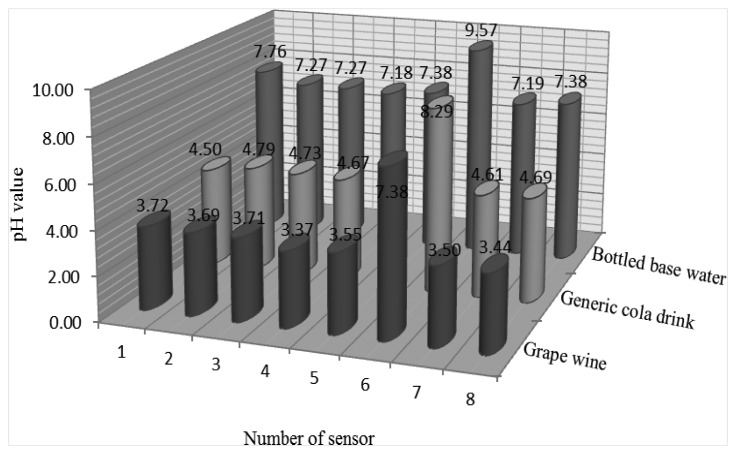
Average of pH measured data of each sensor in grape wine, generic cola drink and bottled water.

The sensitivity of sensor No. 6 is less than 50 mV/pH and lower than that of the others. In this study, we used the RuO_2_ based pH sensor array to repeat fifteen times measurements in grape wine, a generic cola drink and bottled water and to calculate the mean of the pH sensor measurements, which are shown in Figure 9. The pH values of sensor No. 6 in grape wine, generic cola and bottled water are higher than those of the other sensors. These measured data from sensor No. 6 are unusual.

### 3.2. Fault Diagnosis for pH Array Measured Data

We used the measured data from the RuO_2_ sensor array in grape wine, generic cola drink and bottled water [10,13], respectively, to perform the fault diagnosis. Firstly, the Equations (1) and (2) were used to obtain the confidence matrix (D) of the pH sensor array for grape wine, generic cola drink and bottled water measurements. The confidence matrixes (D matrix) are shown as Equations (12), (13) and (14). According to the confidence matrix, the sensors No. 1–5 and sensors No. 7–8 are not consistent with sensor No. 6, because of *d_i6_* (i = 1–5 and 7–8) is extremely small, so the measured data of the 6th sensor will be removed after fault diagnosis, and the pH measured data of sensors 1–5 and 7–8 were used to perform the data fusion in the next step:

(12) $$D=\left(\begin{array}{cccccccc} 1 & 0.993 & 0.931 & 0.902 & 0.954 & 9E-34 & 0.856 & 0.844\\ 0.973 & 1 & 0.867 & 0.512 & 0.926 & 1E-134 & 0.667 & 0.637\\ 0.652 & 0.813 & 1 & 0.125 & 0.985 & 4E-187 & 0.908 & 0.882\\ 0.687 & 0.556 & 0.258 & 1 & 0.349 & 2E-129 & 0.156 & 0.143\\ 0.844 & 0.935 & 0.991 & 0.352 & 1 & 1E-113 & 0894 & 0.874\\ 0.694 & 0.699 & 0.709 & 0.675 & 0.706 & 1 & 0.717 & 0.718\\ 0.954 & 0.971 & 0.995 & 0.856 & 0.991 & 8E-10 & 1 & 1\\ 0.562 & 0.690 & 0.932 & 0.161 & 0.880 & 9E-103 & 0.999 & 1 \end{array}\right)$$

(13) $$D=\left(\begin{array}{cccccccc} 1 & 0.851 & 0.880 & 0.933 & 0.961 & 4E-16 & 0.969 & 0.914\\ 0.868 & 1 & 0.998 & 0.984 & 0.965 & 2E-12 & 0.957 & 0.991\\ 0.835 & 0.997 & 1 & 0.988 & 0.966 & 6E-20 & 0.955 & 0.995\\ 0.935 & 0.982 & 0.992 & 1 & 0.996 & 2E-14 & 0.993 & 0.999\\ 0.940 & 0.940 & 0.963 & 0.994 & 1 & 7E-23 & 0.999 & 0.985\\ 0.393 & 0.444 & 0.438 & 0.426 & 0.418 & 1 & 0.415 & 0.430\\ 0.972 & 0.955 & 0.971 & 0.993 & 0.999 & 8E-14 & 1 & 0.987\\ 0.903 & 0.988 & 0.996 & 0.999 & 0.989 & 2E-16 & 0.983 & 1 \end{array}\right)$$

(14) $$D=\left(\begin{array}{cccccccc} 1 & 0.114 & 0.117 & 0.050 & 0.272 & 2E-13 & 0.054 & 0.192\\ 0.436 & 1 & 1 & 0.975 & 0.958 & 1E-08 & 0.979 & 0.986\\ 0.283 & 1 & 1 & 0.959 & 0.940 & 8E-13 & 0.966 & 0.981\\ 0.554 & 0.987 & 0.986 & 1 & 0.934 & 4E-05 & 1 & 0.961\\ 0.710 & 0.971 & 0.973 & 0.912 & 1 & 1E-05 & 0.919 & 0.995\\ 0.694 & 0.554 & 0.555 & 0.529 & 0.585 & 1 & 0.531 & 0.572\\ 0.424 & 0.984 & 0.983 & 1 & 0.910 & 3E-07 & 1 & 0.949\\ 0.562 & 0.988 & 0.989 & 0.933 & 0.993 & 2E-07 & 0.939 & 1 \end{array}\right)$$

### 3.3. pH Measured Data Used for Data Fusion

The measured data is obtained from in previous references [10,13] and used for the average data fusion, self-adaptive data fusion and coefficient of variance data fusion. The weighted coefficients of average, self-adaptive and coefficient of variance data fusions are obtained from the mean, standard deviation and variance of sensor with the measured data of grape wine, generic cola drink and bottled water and the results are shown in Tables 1, 2 and 3, respectively.

The measured data of sensor No. 6 are uneven and the standard deviation is larger than that of the other sensors. The weighted coefficient equals zero after the fault diagnosis process. The average data fusion has the same weighted coefficients for each sensor and used Equation (5) to obtain the final fusion result shown in Table 4. The different weighted coefficients of self-adaptive data fusion are obtained from Equation (7) and the final fusion result is shown as Table 4. The different weighted coefficients of coefficient of variance data fusion are obtained from Equation (11) and the final fusion result is shown as Table 4. From Table 4, it shows that the data after fault diagnosis and data fusion, are more consistent and close to the measured value of the pH meter.

## 4. Conclusions

In this study, we used the LabVIEW measurement system with a pH sensor array to obtain post-fusion data. Fault diagnosis and data fusion technologies were successfully designed in the LabVIEW program and used for a ruthenium dioxide pH electrode array. The post-fusion measured data were used to study fault diagnosis and data fusion technologies. The experimental results show that one can obtain good fusion results with measured data to perform fault diagnosis before data fusion.

## Figures and Tables

**Table 1.** Comparison of weighted coefficients of ADF, SADF and CVDF with fault diagnosis and obtained from the mean, standard deviation and variance of sensor measured data (sample: grape wine).

**Sample**	**Sensor No. (i)**	**Mean (μ_i_)**	**Standard Deviation (σ_i_)**	**Variance (σ_i_^2^)**	***w****_ADF,i_***(DF)**	***w****_ADF,i_***(FD+FD)**	***w****_SADF,i_***(DF)**	***w****_SADF,i_***(FD+FD)**	***w****_CVDF,i_***(DF)**	***w****_CVDF,i_***(FD+FD)**
Grape wine	1	3.7207	0.150498	0.022650	0.125	0.142857	0.046318	0.046328	0.088915	0.090007
	2	3.6893	0.074591	0.005564	0.125	0.142857	0.188555	0.188596	0.177888	0.180072
	3	3.7093	0.063185	0.003992	0.125	0.142857	0.262772	0.262829	0.211138	0.213730
	4	3.3733	0.078619	0.006181	0.125	0.142857	0.169729	0.169766	0.154318	0.156213
	5	3.5540	0.079624	0.006340	0.125	0.142857	0.165471	0.165507	0.160531	0.162502
	6	7.3767	2.187561	4.785424	0.125	0	0.000219	0	0.012128	0
	7	3.4993	0.276831	0.076635	0.125	0.142857	0.013689	0.013692	0.045463	0.046021
	8	3.4420	0.082739	0.006846	0.125	0.142857	0.153247	0.153281	0.149619	0.151456

**Table 2.** Comparison of weighted coefficients of ADF, SADF and CVDF with fault diagnosis and obtained from the mean, standard deviation and variance of sensor measured data (sample: generic cola drink).

**Sample**	**Sensor No. (i)**	**Mean (μ_i_)**	**Standard Deviation (σ_i_)**	**Variance (σ_i_^2^)**	***w****_ADF,i_***(DF)**	***w****_ADF,i_***(FD+FD)**	***w****_SADF,i_***(DF)**	***w****_SADF,i_***(FD+FD)**	***w****_CVDF,i_***(DF)**	***w****_CVDF,i_***(FD+FD)**
Generic cola drink	1	4.50	0.226716	0.051400	0.125	0.142857	0.126593	0.127022	0.125977	0.130926
	2	4.75	0.240143	0.057669	0.125	0.142857	0.112833	0.113215	0.125647	0.130583
	3	4.72	0.189882	0.036055	0.125	0.142857	0.180470	0.181082	0.157924	0.164128
	4	4.67	0.229031	0.052455	0.125	0.142857	0.124047	0.124467	0.129285	0.134364
	5	4.63	0.183796	0.033781	0.125	0.142857	0.192620	0.193273	0.159769	0.166046
	6	8.30	1.388524	1.927998	0.125	0	0.003375	0	0.037801	0
	7	4.61	0.235992	0.055692	0.125	0.142857	0.116837	0.117232	0.124001	0.128873
	8	4.69	0.213146	0.045431	0.125	0.142857	0.143225	0.143710	0.139595	0145079

**Table 3.** Comparison of weighted coefficients of ADF, SADF and CVDF with fault diagnosis and obtained from the mean, standard deviation and variance of sensor measured data (sample: bottled water).

**Sample**	**Sensor No. (i)**	**Mean (μ_i_)**	**Standard Deviation (σ_i_)**	**Variance (σ_i_^2^)**	***w****_ADF,i_***(DF)**	***w****_ADF,i_***(FD+FD)**	***w****_SADF,i_***(DF)**	***w****_SADF,i_***(FD+FD)**	***w****_CVDF,i_***(DF)**	***w****_CVDF,i_***(FD+FD)**
Bottled water	1	7.76	0.118454	0.014031	0.125	0.142857	0.322554	0.323866	0.227003	0.234350
	2	7.27	0.190938	0.036457	0.125	0.142857	0.124143	0.124648	0.131902	0.136171
	3	7.27	0.153598	0.023592	0.125	0.142857	0.191837	0.192617	0.164028	0.169337
	4	7.18	0.266104	0.070811	0.125	0.142857	0.063915	0.064175	0.093352	0.096551
	5	7.38	0.229952	0.052878	0.125	0.142857	0.085591	0.085939	0.111190	0.114789
	6	9.57	1.057183	1.117635	0.125	0	0.004050	0	0.031351	0
	7	7.19	0.218660	0.047812	0.125	0.142857	0.094660	0.095044	0.113933	0.117620
	8	7.33	0.199910	0.039964	0.125	0.142857	0.113250	0.113710	0.127068	0.131181

**Table 4.** Comparison of fusion results of ADF, SADF and CVDF with measured data of grape wine, generic cola drink and bottled water with fault diagnosis.

	**Methods**	**Average Data Fusion (ADF)**	**Self-Adaptive Data Fusion (SADF)**	**Coefficient of Variance Data Fusion (CVDF)**

	**Samples**			
Data Fusion (DF)	Grape wine	4.05	3.58	3.62
	Generic cola drink	5.11	4.67	4.79
	Bottled water	7.62	7.44	7.46

Fault Diagnosis + Data Fusion (FD+DF)	Grape wine	3.57	3.58	3.58
	Generic cola drink	4.65	4.65	4.65
	Bottled water	7.34	7.43	7.39

Commercial pH Meter	Grape wine		3.61	
	Generic cola drink		4.24	
	Bottled water		7.32	
